# Nebulisation of synthetic lamellar lipids mitigates radiation-induced lung injury in a large animal model

**DOI:** 10.1038/s41598-018-31559-3

**Published:** 2018-09-06

**Authors:** David Collie, John T. Murchison, Steven H. Wright, Alec McLean, Lynsey Howard, Jorge del-Pozo, Sionagh Smith, Gerry McLachlan, Jessica Lawrence, Elaine Kay, Tobias Schwarz, Magdalena Parys

**Affiliations:** 1 The Roslin Institute and Royal (Dick) School of Veterinary Studies, University of Edinburgh, Edinburgh, United Kingdom; 2 Edinburgh Imaging, College of Medicine and Veterinary Medicine, University of Edinburgh, Edinburgh, Scotland United Kingdom; 3 Lamellar Biomedical Ltd, Eurocentral, Holytown, Scotland United Kingdom

## Abstract

Methods to protect against radiation-induced lung injury (RILI) will facilitate the development of more effective radio-therapeutic protocols for lung cancer and may provide the means to protect the wider population in the event of a deliberate or accidental nuclear or radiological event. We hypothesised that supplementing lipid membranes through nebulization of synthetic lamellar lipids would mitigate RILI. Following pre-treatment with either nebulised lamellar lipids or saline, anaesthetised sheep were prescribed fractionated radiotherapy (30 Gray (Gy) total dose in five 6 Gy fractions at 3–4 days intervals) to a defined unilateral lung volume. Gross pathology in radio-exposed lung 37 days after the first radiation treatment was consistent between treatment groups and consisted of deep red congestion evident on the pleural surface and firmness on palpation. Consistent histopathological features in radio-exposed lung were subpleural, periarteriolar and peribronchial intra-alveolar oedema, alveolar fibrosis, interstitial pneumonia and type II pneumocyte hyperplasia. The synthetic lamellar lipids abrogated radiation-induced alveolar fibrosis and reduced alpha-smooth muscle actin (ASMA) expression in radio-exposed lung compared to saline treated sheep. Administration of synthetic lamellar lipids was also associated with an increased number of cells expressing dendritic cell-lysosomal associated membrane protein throughout the lung.

## Introduction

Radiation therapy is prescribed in over 50% of patients receiving cancer treatment. Radiation-induced lung injury (RILI), namely pneumonitis and pulmonary fibrosis, can follow radiation treatment to thoracic structures, chest wall, and lower neck, either because lung is part of the target volume or due to its proximity to the tumour target. Pneumonitis follows early after treatment and may be reversible however fibrosis is delayed and irreversible^1,2^. Rates of RILI can be as high as 70–80% in high dose regions of lung^3^. As there are no reliable predictive models to provide the likelihood or severity of RILI prior to radiation therapy, risk is reduced through dose constraints. Indeed, the risk of developing radiation pneumonitis shapes current recommendations in human planning to minimize the volume of lung receiving 20 Gy (V20) to less than 30%^4–7^. Other factors that contribute to pneumonitis such as age, concurrent chemotherapy and baseline CT findings are difficult to control, thus radiation dose constraints are important when weighing the risk of adverse events^8^. However, such dose limitation may compromise tumour control. New approaches to mitigate RILI are urgently needed to improve quality of life and long-term outcomes in cancer survivors^9–14^. Such approaches could also serve as a valuable resource for treating mass casualties in the context of a deliberate, or accidental, nuclear or radiological event^15^.

Instantaneous lung tissue injury occurs following exposure to ionising radiation, predominantly resulting in indirect radiation damage from the generation of highly reactive free radicals. Radiation induces DNA base damage, single and double-strand breaks, protein cross-linking and fragmentation, and membrane lipid peroxidation, which leads to both acute alveolar epithelial and endothelial (capillary) damage. Whilst maintaining an intact plasma membrane is essential to osmotic homeostasis and cell survival, its component phospholipids are at particular risk of injury from excessive reactive oxygen species (ROS) generation^16^. Such ROS cause peroxidation of membrane lipids and increase the ionic permeability of the membrane. The energy cost associated with trying to maintain ionic concentration gradients in the face of such altered structure and function can severely deplete cytoplasmic ATP stores, leading to further generation of cellular ROS, and eventual cell death and necrosis.

Surfactant is also susceptible to damage by ROS^17–19^, leading to a decrease in its surface activity as a consequence of alterations to component lipids and proteins. Such damage promotes heterogeneous airway collapse and increased lung stiffness, further stressing interspersed areas of normal lung parenchyma and propagating injury and inflammation.

Type II pneumocytes are considered a key element in the pathogenesis of RILI^20,21^. Lamellar bodies in type II pneumocyte cells are depleted early after lung irradiation, a change coupled with increased surfactant levels in alveolar lavage fluid^22^. A reduced rate of surfactant turnover in mice following lung irradiation is noted up to six weeks following exposure^23^.

Surfactant is used to prevent and treat respiratory distress syndrome (RDS) in infants where a primary deficiency occurs as a consequence of lung immaturity^24^. Indeed, early administration of surfactant has been shown to suppress serum levels of TGF-β1 when combined with mechanical ventilation, improving treatment efficacy of RDS^25^. In addition surfactant administration has been proposed for ARDS/ALI, where surfactant function is impaired^26^. We proposed that the rationale driving surfactant therapy in these instances could apply equally to the administration of synthetic lamellar lipids for mitigating RILI, where pneumocyte injury occurs early. In this study we sought to determine whether synthetic lamellar lipids (LAMELLASOME^TM^, Lamellar Biomedical Ltd, Bellshill, Scotland, UK) delivered prior to irradiation could reduce RILI in a novel large animal system used to model the effects of radiotherapy on the lung.

## Materials and Methods

### Ethics statement

Experimental protocols were reviewed and approved by the Roslin Institute Animal Welfare and Ethics Committee. All experiments were performed in accordance with the relevant guidelines and regulations relating to the provisions of the Animals (Scientific Procedures) Act 1986.

### Animals

Twelve commercially sourced adult Shetland sheep (bodyweight: 38.5 kg [33.0–43.0] median [range]; 6 female and 6 castrated male) were included in this study. Animals were housed for the duration of the study and otherwise maintained according to the code of practice for the housing and care of animals bred, supplied and used for scientific purposes^27^. The sheep were treated with an anthelminthic before the study began and were randomly allocated to one of two sex-matched treatment groups.

### Experimental design

In order to confirm the absence of pre-existing pulmonary disease and collect baseline samples against which to judge change within-animals, preliminary baseline examination (BBr1) involving bronchoscopic visualisation, bronchoalveolar lavage and bronchial brush biopsy under gaseous anaesthesia was conducted. Two additional baseline examinations (BBr2 & BBr3) involving bronchial brush biopsy sampling were conducted at fortnightly intervals along with measurements of body weight and rectal temperature. At least two weeks after the last baseline assessment (BBr3) the sheep were re-anaesthetised to enable acquisition of thoracic computed tomography (CT) images for subsequent radiation treatment planning. Radiation was prescribed to a defined volume of left lung and administered as detailed below. Nebulised synthetic lamellar lipids (LAMELLASOME^TM^; Lamellar Biomedical Ltd, Bellshill, United Kingdom), or saline aerosol, was administered prior to each radiation treatment as described below. Following the last radiation treatment (t0) sheep were monitored for any evidence of adverse effect. At t0 + 11d and t0 + 21d the sheep were re-anaesthetised and subjected to bronchial brush biopsy in the same manner as during the preliminary baseline evaluation. At t0 + 23d the sheep were euthanized by overdose of anaesthetic and presented for necropsy examination.

### Aerosol delivery

Prior to each radiation treatment conscious sheep were restrained in a holding crate to facilitate the placement of a face mask connected to a nebuliser (eFlow rapid nebuliser system, Pari GmbH, Germany). Nebulisation of saline, or LAMELLASOME^TM^ (20 mg/ml), was conducted over a 15 minute period and the interval between nebulisation and radiotherapy ranged from 70 to 110 mins.

### Anaesthesia

Induction of anaesthesia was achieved using an intravenous injection of 6–8 mg/kg propofol (Fresenius propofol, 1%, Fresenius Kabi Ltd) and thereafter sheep were maintained under isoflurane anaesthesia using positive pressure ventilation (Model 708; Harvard Apparatus, Millis, MA).

### Image acquisition and conformal radiation treatment

Vacuum-formable mattresses facilitated reproducible sternal positioning for thoracic CT scan (Somatom Volume Zoom, Siemens, Germany) and radiation therapy (Linear Accelerator, Varian Clinac 2100 C/D). Images were obtained during anaesthesia with breath-hold to avoid motion artefact. Treatment planning software (Eclipse v11.0, Varian Medical Systems Inc, Palo Alto, CA) was employed to delineate structures and enable conformal 3-dimensional 6MV photon plan design by a board certified veterinary radiation oncologist blinded to the treatment groups. Organs at risk (OAR) were outlined to permit dose calculations and consisted of left lung, right lung, left mainstem bronchus, right mainstem bronchus, heart, oesophagus, trachea, spinal cord, reticulum, rumen, abomasum, and omasum. A clinical target volume (CTV) was manually created in the left caudal lung lobe starting at a level approximately 6 cm caudal to the level of the tracheal bifurcation, such that the medial border was 1 cm away from midline and the lateral border 0.5 cm away from the lateral thoracic body wall. A planning target volume (PTV) was created following 3 cm expansion of the CTV in all directions, confined within the left lung. At least 0.5 cm between the medial PTV edge and midline was required to ensure the mean dose scattered to the right lung was less than 1 Gy. The radiation prescription consisted of 5 fractions of 6 Gy to isocentre (central point defined in the target volume through which radiation beams pass) within the PTV to a total dose of 30 Gy with each fraction to be administered at 3- or 4-day intervals. In order for the plan to be approved, minimum dose to CTV and PTV were required to be 26 Gy and 25 Gy, respectively. Due to the lack of solid mass within air-filled lung, dose heterogeneity was permitted provided 95% of the PTV received at least 27 Gy and the mean dose was 29–30 Gy. The volume of lung receiving 20 Gy or higher (V20) was limited to 30%. Additional dose constraints determined prior to the study to avoid clinical toxicity included constraints to reticulum (V20 < 20%), rumen (V20 < 20%), abomasum (V15 < 20%), and liver (V30 < 20%). Maximum dose for any OAR was set at 33 Gy. To enable radiation treatment, sheep were anaesthetized and positioning verified with orthogonal on-board portal images. Breath-hold was implemented for each radiation treatment field.

### Bronchoalveolar lavage

The bronchoscope (Model FG-15W; Pentax UK Ltd.) was wedged in the segmental bronchus of the right apical lobe. Two 20 ml aliquots of PBS were used to collect bronchoalveolar lavage fluid (BALF) from this lung segment and samples were processed and assessed using previously described methodology^28^.

### Bronchial brush biopsy

On each occasion of baseline assessment three bronchial brush biopsy samples were derived from each of three separate areas of the lung (n = 9 total per animal). Samples were taken from bronchi within the left caudal diaphragmatic lung lobe (LCD), the right caudal diaphragmatic lung lobe (RCD), and also from bronchi within areas of the anterior right lung. Considerable care was taken (through manual mapping and reference to video recordings at each brush biopsy time point) to avoid sampling any area of bronchial epithelium that had previously been subject to bronchial brush biopsy. At t0 + 11d and t0 + 21d the sheep were subject to bronchial brush biopsy in the same manner as during the preliminary baseline evaluations.

### Necropsy

Following euthanasia by intravenous injection of barbiturate, the heart and lungs were removed from the carcase following standard necropsy protocols. The pulmonary circulation was perfused via the pulmonary artery with 2 litres of PBS before the heart was dissected away. The lungs were then photographed before being presented for further processing. Lung tissue was fixed over seven days by airway instillation of 10% neutral buffered formalin in a manner similar to that previously described^29^.

### Gross tissue sampling

After fixation each lung was carefully sliced along the transverse plane, starting at the caudal pole of each diaphragmatic lobe, into fifteen 1 cm thick tissue slices. These slices were then arranged in order for photographing prior to a representative tissue block from each contiguous slice being selected and carefully dissected from surrounding lung tissue. A further photographic image of the slices with their selected blocks *in situ* was captured to document the spatial origin of each block. This latter step was a necessary prerequisite to registering the position of each block with respect to the radiation field through reference to the axial CT images previously collected from the same animals. The isocentre PTV was determined in x, y, and z coordinates from the level of the tracheal bifurcation. The cranial and caudal edges of the PTV were measured from isocentre to provide precise mapping to the radiation treatment plan. Tissue blocks were then submitted for standard histological processing and paraffin embedding.

### Block selection

The formalin-fixed paraffin embedded (FFPE) tissue block from the left caudal diaphragmatic lung that represented the isocentre of the planning target volume was identified and selected, as was the corresponding block from the right contralateral control lung. These blocks were thenceforth labelled LL_Post and RL_Post respectively (“LL” and “RL” for left and right lung respectively, and “Post” for posterior). A further FFPE tissue block was sourced anterior (14.5 mm [9.7–23.0]) to the cranial margin of the PTV (LL_Ant), as well as its corresponding block from the right contralateral control lung (RL_Ant).

### Histochemical and immunohistochemical staining

Sections cut from the above blocks were stained with haematoxylin–eosin (H&E) and Picrosirius Red, as well as being immunostained with antibodies specific for the following antigens – alpha-smooth muscle actin (ASMA), dendritic cell-lysosomal associated membrane protein (DC-LAMP), and Ki67 protein (a marker for cell proliferation). All slides were stained using standard immunohistochemistry methods with endogenous peroxidase blocked using 3% H_2_O_2_ in methanol and heat-induced antigen retrieval performed using 10 mM citrate buffer pH6.0. Details of the IHC protocols are provided in supplementary information (Supplementary Methods).

### Bronchial brush cytokine expression

Bronchial brush biopsy specimens were collected using cytology Brushes (Conmed Endoscopic Technologies 152 R) agitated into 1 ml of cold sterile PBS (Sigma D8537) through 200 µl wide orifice pipette tips (Star Lab E1011-8000) and centrifuged at 10,000 g for 5 minutes. Pellets were resuspended in RLT buffer (Qiagen 74106) containing 1% β mercaptoethanol and stored at −80 °C until extraction. All samples were run through Qiashredder columns (Qiagen 79656) and RNA extractions were performed using RNeasy mini kit (Qiagen 74106) with DNase treatment using RNase free DNase set (Qiagen 79254). RNA was quantified on Nanodrop and quality checked on Agilent Tapestation with RNA screentape (Agilent 5067–5576). cDNA was prepared from 400 ng RNA with Transcriptor First Strand cDNA Synthesis kit (Roche 04 896 866 001) using random hexamer primers. Quantitative Real Time PCR was performed using the Lightcycler 480 with 2.5 µl cDNA in LightCycler 480 Sybr Green I Master (Roche 04 887 352 001) and specific primers. Advanced relative quantification was calculated using Lightcycler 480 SW1.5 programme. Standard curves for each gene were generated from pooled ovine alveolar macrophage cDNA. Melt curve analysis showed a single peak for all samples. PCR efficiency was in the range of 1.8 to 2.1. qPCR conditions and referenced primer sets^30–33^ are provided in supplementary information (Tables S1 and S2 Supplementary Methods).

### Semiquantitative histopathological evaluation

One pathologist (SHS) examined an unblinded, reduced subset of representative samples (i.e. saline treated only) to obtain preliminary histopathology results to inform further analysis, whereas the pathologist who examined all of the datasets (JDP) was blinded to the results of the preliminary analysis and to the source of the slides. All H&E stained sections were scanned on a whole slide scanner (Nanozoomer, Hamamatsu, Japan) to acquire whole slide images (WSI) at x40 magnification. These sections were then subject to detailed examination by JDP. Following an initial appraisal in which principal pathologic features were identified, a semi-quantitative scoring system was developed to capture the incidence and extent of each feature amongst the different sections. Briefly, to score lesions for feature severity, each section was allocated a score ranging from 0 (absent), 1 (mild), 2 (moderate), to 3 (severe). Features noted included intra-alveolar macrophages, oedema, or fibrin, interstitial pneumonia, periarteriolar oedema or inflammation, and peribronchial or peribronchiolar inflammation. Additionally, fibrosis was scored by allocation of an estimated % surface involved. Type II pneumocyte cell hyperplasia and atypia, and epithelial atypia were scored qualitatively (i.e. presence or absence).

### Quantitative histological analyses

Within ImageJ, the NDPITools custom extract to TIFF/mosaic plugin was used to extract each ndpi image file from whole slide images (WSI) to multiple TIFF images. H&E stained sections were extracted at x20 resolution, and the remaining WSIs at x40 resolution. Image fields containing parenchyma (including airways no larger than respiratory bronchioles) were then manually selected from a random selection of these extracted files. These files were then converted to OME-TIFF using an ImageJ recursiveTiffConvert macro to engage the Bio-Formats exporter function. Parenchymal x40 OME-TIFF files were batch processed using macros employing the colour devolution plugin for detecting the area of red-stained collagen in Picrosirius Red-stained sections, and DAB stain in ASMA, DC-LAMP and Ki67 immunostained sections. For measurements on Picrosirius Red-stained sections, and DAB stain in ASMA, and DC-LAMP immunostained sections sample size was considered acceptable if the standard error for measurements fell below 5% of the mean value for that measurement. In the six sections where this condition was not met, the standard error ranged from 5.0 to 7.3% of the mean. As the scarcity of Ki67-stained cells in control lung sections meant that the 5% limit could not be achieved for the majority of sections, a pragmatic decision on sampling was taken. In this latter regard the number of fields examined per section ranged from 172–198.

### Statistical analyses

Continuous data was assessed for normality of distribution using a Kolmogorov_Smirnoff test. Where necessary data transformation was applied to normalise the distribution, and where such transformation failed, a rank-order transformation was applied prior to subsequent evaluation. Ordinal data from semi-quantitative histopathological analyses was rank-order transformed.

For repeated measures data (clinical and cytokine data) a General Linear Model was fitted in which responses in radio-exposed and contralateral control lung segments were evaluated with respect to time, and the experimental treatment (LMS, SAL). Sheep identity, nested within treatment, was considered a random factor in the design. For the analysis of quantitative histopathological and immunohistochemical data a General Linear Model two-way analysis of variance (ANOVA) was conducted on the influence of the two independent variables (Lung, Treatment) on the variable in question. Lung included four levels (LL_Ant, LL_Post, RL_Ant, RL_Post), and Treatment two levels (LMS, SAL). A further analysis was conducted in which the influence of sheep gender (MN, F) was included. Significance was set at P < 0.05.

## Results

### Clinical

No adverse effects were noted either as a consequence of aerosol delivery, or secondary to radiation treatment. At every time point sheep were weighed and rectal temperature recorded. Whilst no loss of weight was experienced by either group, the saline-treated sheep experienced a minor (ie remaining within normal range), but statistically significant, increase in temperature at baseline 2 & 3 (data not shown). The possibility that this increase could have heralded an otherwise subclinical phenomenon prompted use of the first evaluation as the baseline time point.

### Bronchial brush biopsy cytokine expression

Neither Treatment nor Time had a significant effect on IL1beta or TGFbeta expression levels in samples derived from RCD or LCD, nor in relation to IL8 expression levels in samples derived from RCD. However, for samples derived from LCD, Time had a significant effect on Log_10_ IL8 expression levels (p = 0.030), reflecting a decrease in expression at Time point 4 (data not shown). Treatment did not have a significant effect.

### Gross necropsy observations

**Figure 1:**
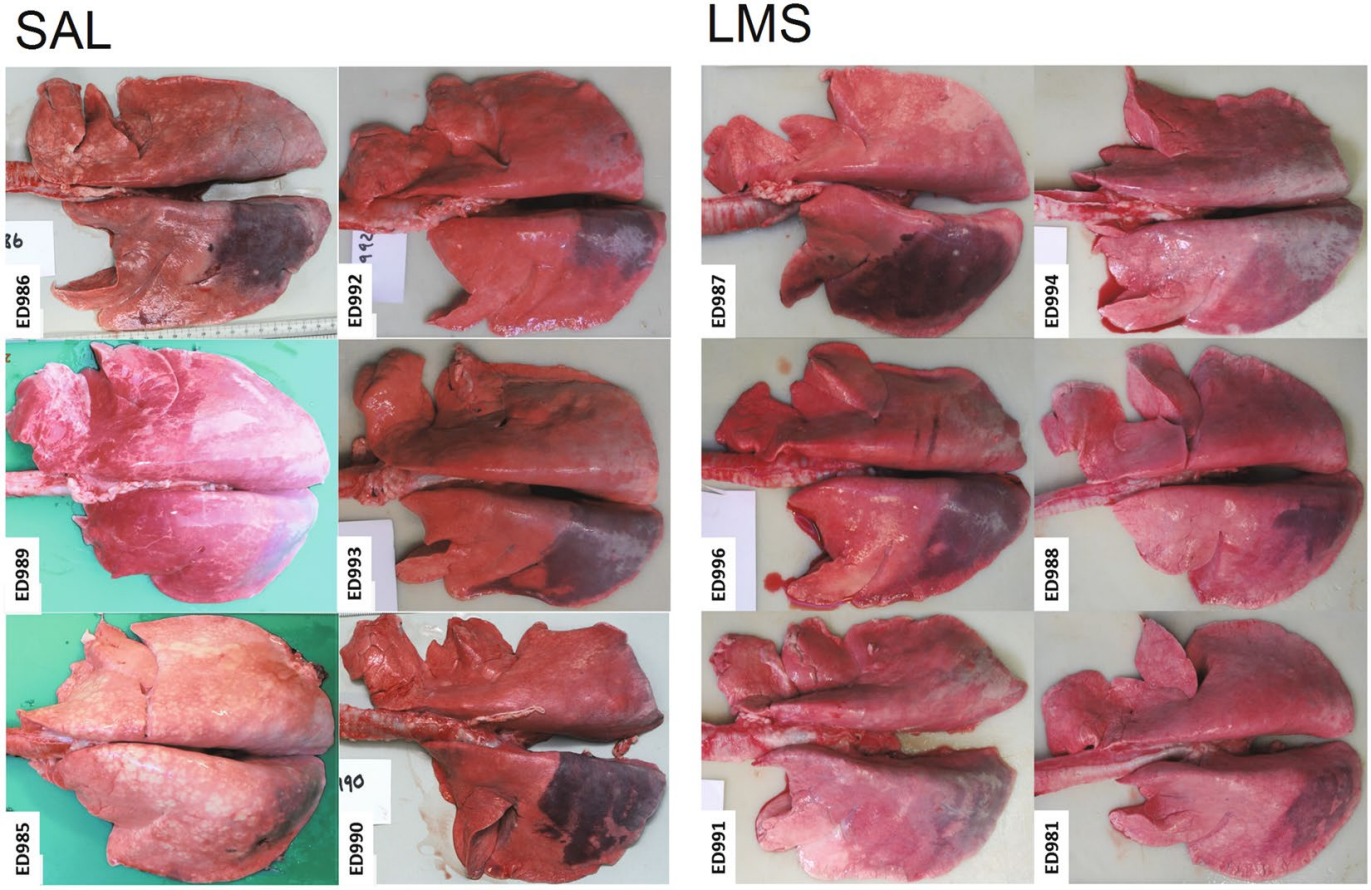
Gross pathological features associated with RILI in sheep. Photographs of lungs removed from sheep treated with nebulised saline (SAL) or LAMELLASOME^TM^ (LMS) prior to each of 5 fractions of 6 Gy radiation delivered to the left caudal diaphragmatic lobe at 3–4 day intervals. The area of dark red pleural discolouration which reflects the margins of the PTV was clearly evident in all the lungs. Whilst there was no substantial difference in the gross appearance of the lungs from each group there was clearly some sheep to sheep variation in the nature and extent of the radiation-induced discolouration.

At necropsy, the pleural surface covering the PTV was easily identifiable as a consequence of dark red discolouration (Fig. 1). The underlying radio-exposed lung parenchyma was firmer on palpation and, when investigated in one instance, the affected lung volume failed to inflate properly.

### Histopathological evaluation

**Figure 2:**
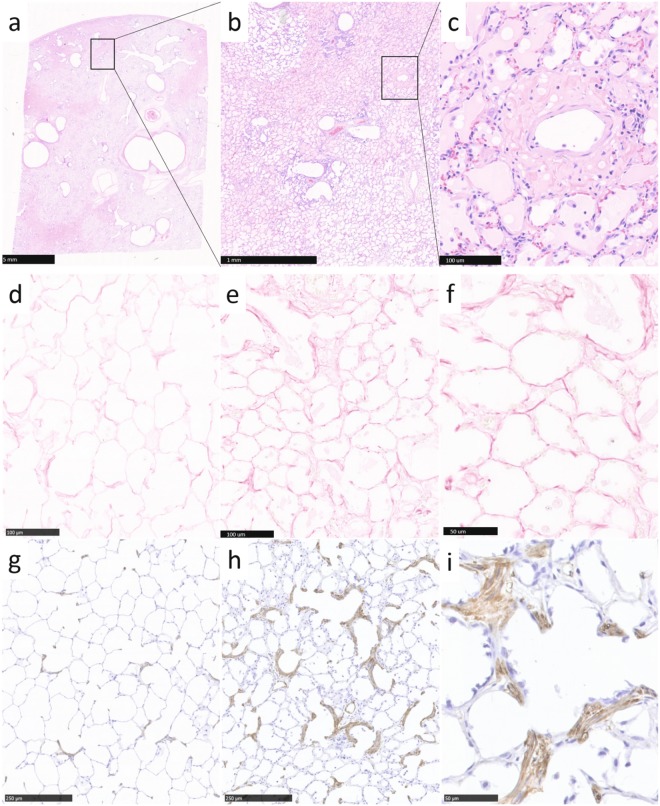
Histopathological features associated with RILI in sheep. Panels (a–c) photomicrographs of an H&E-stained section illustrating the oedema that arises as a consequence of radiation exposure to the sheep lung. Oedema could be appreciated macroscopically (**a**) and was frequently found in the subpleural region (scale bar 5 mm). The borders between areas of oedema and aerated lung were often sharply demarcated (**b**) (scale bar 1 mm). Perivascular oedema was also recognised (**c**) (scale bar 100 μm). Panels (d–f) photomicrographs of picrosirius red-stained sections highlighting collagen deposition. Panels (e) and (f) are section images from radio-exposed lung (scale bars 100 and 50 μm respectively) and (**d**) from the contralateral control lung (scale bar 100 μm). Radiation exposure was associated with an increase in the area percentage of collagen. Panels (g–i) photomicrographs highlighting the expression of ASMA in radio-exposed lung (**h** and **i**, scale bars 250 and 50 μm respectively), and from the contralateral control lung (**g**) (scale bar 250 μm). ASMA expression in the non-radio-exposed lung was sparse and found in association with the alveolar ducts, both at the septal tips and in the alveolar walls. Radiation exposure led to an increase in ASMA expression in these areas.

The main parenchymal abnormalities noted in all irradiated lungs were subpleural. There was periarteriolar and peribronchial oedema (Fig. 2; panels a–c) characterized by periarteriolar and intra-alveolar accumulation of homogeneous, proteinaceous material, with occasional fibrillar material (fibrin), and aggregates of eosinophilic smudged material (fibrin). These areas also featured increased numbers of intra-alveolar macrophages, often containing foamy cytoplasm. Alveolar fibrosis was characterized by mild thickening of alveolar walls by deposition of pale eosinophilic fibrillary material and there was interstitial pneumonia comprising infiltration of alveolar walls by small numbers of lymphocytes and plasma cells, scattered type II pneumocyte hyperplasia and occasional atypia. The atypia was characterized by increased nuclear:cytoplasmic ratio, apical blebbing, mild pleomorphism, and nuclei with finely stippled chromatin and small nucleoli. Airway changes in radio-exposed lung included mild submucosal infiltration by lymphocytes and plasma cells, and bronchial and bronchiolar epithelial atypia similar to that described for type II pneumocytes. Other histopathological abnormalities noted in a small number of sections involved parasite granulomas which were interpreted as incidental and unrelated to the treatment.

**Figure 3:**
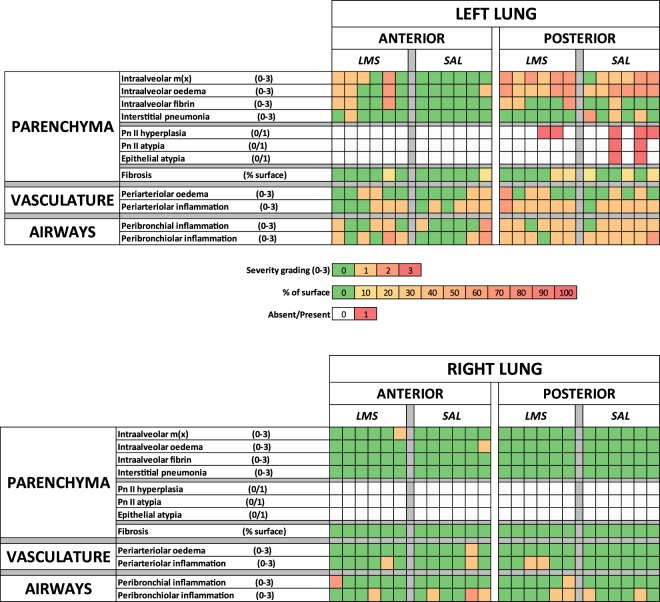
Histopathology quantitation. Heatmap representation of the results of blinded semiquantitative analysis of the principal histopathological features associated with radiation exposure in the sheep lung. The upper panel reports the findings relating to the left lung, and the lower, the right lung. Within each lung the areas of assessment were further subdivided into posterior and anterior, reflecting the origin of the blocks submitted for assessment. Blocks derived from the posterior volume of the left lung lay within the PTV and were directly exposed to radiation, whilst blocks derived from the anterior volume lay proximal to the cranial margins of the PTV and should not have been directly exposed to radiation. Equally blocks from the right lung were identified as contralateral controls for the left lung samples. The panels are further subdivided according to the treatments, LAMELLASOME^TM^ (LMS) or saline (SAL), with the colour of each cell representing the semiquantitative scoring of the histopathological features listed to the left hand side of each row in the heatmap.

The results of the histopathological evaluation are depicted in Fig. 3. Lung irradiation was associated with a statistically significant increase in intra-alveolar oedema (P = 0.000), macrophages (P = 0.000), and fibrin (P = 0.022), interstitial infiltrates of lymphocytes (P = 0.018), periarteriolar oedema (P = 0.041) and inflammation (P = 0.000), perivascular infiltrates of plasma cells (P = 0.010) and lymphocytes (P = 0.002), type II pneumocyte hyperplasia (P = 0.000) and parenchymal fibrosis (P = 0.025). LAMELLASOME^TM^ treatment was associated with a statistically significant increase in the number of intra-alveolar macrophages (P = 0.004), and the amount of intra-alveolar fibrin (P = 0.022).

### Quantitative Histochemistry and Immunohistochemistry

#### Alveolar fibrosis

Picrosirius red staining in non-radio-exposed lung was evident throughout the alveolar septa (Fig. 2; panel (d)). The most intense staining took the form of wavy filiform linear deposits of variable length and thickness. The septal crests and alveolar walls abutting alveolar ducts often featured more diffuse staining where individual fibres seemed teased apart into subunit fibrils. In radio-exposed lung, fibres present in the thickened alveolar septa similarly appeared teased apart giving a subjective impression of more abundant staining (Fig. 2; panels (e) & (f)). Within sections from radio-exposed lung there was often substantial variation in the extent of staining between fields, with some areas appearing identical to those from control lung sections.

**Figure 4:**
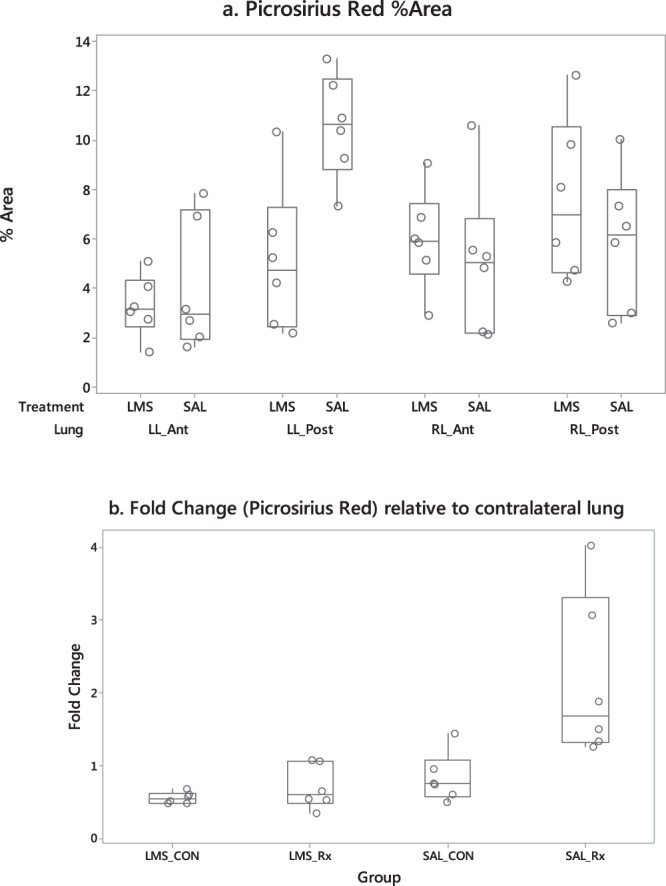
Quantification of Picrosirius Red staining. (**a**) Boxplot depicting data relating to the percentage area of collagen present in Picrosirius red-stained lung parenchymal sections derived from the lungs of sheep previously exposed to radiation. Sections were derived from the left caudal diaphragmatic lung (LL_Post; representing the isocentre of the PTV), its contralateral control (RL_Post), a within-lung control sourced anterior to the cranial margin of the PTV (LL_Ant), as well as its corresponding block from the right contralateral control lung (RL_Ant). Boxplots are further categorised according to the treatment (SAL or LMS) that the sheep received prior to radiation exposure. (**b**) Boxplot depicting the fold change in percentage area of collagen in sections derived from the left lung, relative to the right lung contralateral control sections paired within animal (LL/RL). LMS_CON and SAL_CON are the fold changes between LL_Ant and RL_Ant, and LMS_Rx and SAL_Rx are the fold changes between LL_Post and RL_Post. Only the sheep pre-treated with saline demonstrated a significant increase in fold change in the left (radio-exposed) lung relative to the right contralateral control lung.

The %Area of lung parenchyma occupied by collagen is depicted in Fig. 4a. The highest values are found in saline-treated radio-exposed lung. A Two-way ANOVA (Table 1) indicated that there was a significant difference in the mean percentage collagen between the different lung segments (P = 0.002). Whilst the effect for treatment was not significant (P = 0.227) the interaction effect was significant (P = 0.008), indicating that the impact of Lung depends on the Treatment. Examining the fold change in %Area of collagen found in radio-exposed lung relative to its contralateral control for sheep pre-treated with saline (SAL_Rx) or LAMELLASOME^TM^ (LMS_Rx), and in non-radio-exposed lung relative to its contralateral control for sheep pre-treated with saline (SAL_CON) or LAMELLASOME^TM^ (LMS_CON), determines that the fold change seen in the SAL_Rx group (Fig. 4b) significantly exceeds that seen in any other group (P = 0.001).

**Table 1:** Summary of two-way ANOVA statistics examining the influence of two factors, Lung (with four levels: LL_Ant, LL_Post, RL_Ant, RL_Post), Treatment (with two levels: LMS, SAL), and their interaction (Lung*Treatment) (total degrees of freedom = 40) on % Area collagen, % Area ASMA, % Area DC-LAMP stain, the number and size of DC-LAMP positive particles, the nearest neighbour distance (NND) between DC-LAMP positive particles, and the number of Ki67 positive particles. The F ratios and P values are depicted in the factor columns, and the fitted means (SE mean) for each factor level is given in the level columns. For clarity, the latter are only shown where the relevant factor effect is significant.

Variable		Factor	Levels				Factor	Levels		Interaction
		Lung	LL_Ant	LL_Post	RL_Ant	RL_Post	Treatment	LMS	SAL	Lung*Treatment
% Area collagen	F ratio	5.72	3.685 (0.750)	7.787 (0.750)	5.562 (0.750)	6.751 (0.750)	1.51			4.53
	P	0.002					0.227			0.008
% Area ASMA	F ratio	1.26					28.45	0.1060 (0.0507)	0.4882 (0.0507)	2.58
	P	0.303					0.000			0.067
% Area DC-LAMP stain	F ratio	0.31					3.33			0.39
	P	0.820					0.076			0.759
Number of DC-Lamp positive particles	F ratio	0.17					4.19	61.73 (3.56)	51.43 (3.56)	1.35
	P	0.918					0.047			0.273
Size of DC-LAMP positive particles	F ratio	10.76	2.8953 (0.0174)	2.9649 (0.0174)	2.8327 (0.0174)	2.8599 (0.0174)	0.00			1.75
	P	0.000					0.966			0.170
DC-LAMP NND	F ratio	47.92	17.83 (1.95)	8.67 (1.95)	36.25 (1.95)	35.25 (1.95)	0.36			5.66
	P	0.000					0.554			0.002
Number of Ki67 positive particles	F ratio	7.75	1.1208 (0.0755)	1.3704 (0.0755)	0.9011 (0.0755)	0.9589 (0.0755)	1.55			3.19
	P	0.000					0.221			0.034

#### ASMA expression

**Figure 5:**
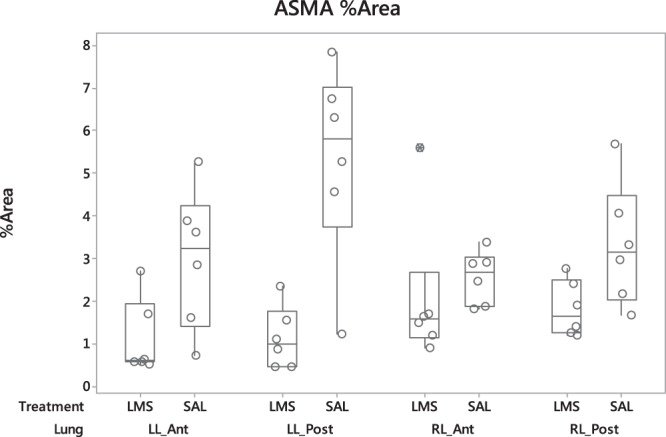
Quantification of ASMA staining. Boxplot depicting data relating to the percentage area of ASMA present in lung parenchymal sections derived from the lungs of sheep previously exposed to radiation. The source of the sections is as described in the legend for Fig. 4, and in the material and methods.

ASMA expression in non-radio-exposed lung was evident at the tips of secondary septal crests abutting alveolar ducts, as well as within alveolar walls similarly adjacent to ducts (Fig. 2; panel (g)). The same general pattern of expression, but increased in area, was evident for radio-exposed lung (Fig. 2; panels (h) & (i)). The average percentage area of ASMA positivity is depicted in Fig. 5. Two-way ANOVA (Table 1) indicated no significant lung effect (P = 0.303), a highly significant treatment effect (P = 0.000), and a non-significant interaction effect (P = 0.067). There was a significant positive association between the %Area occupied by collagen (Picrosirius red) and that occupied by ASMA in both groups (P = 0.029 for SAL, and P = 0.009 for LMS) (data not shown).

#### DC-LAMP expression

**Figure 6:**
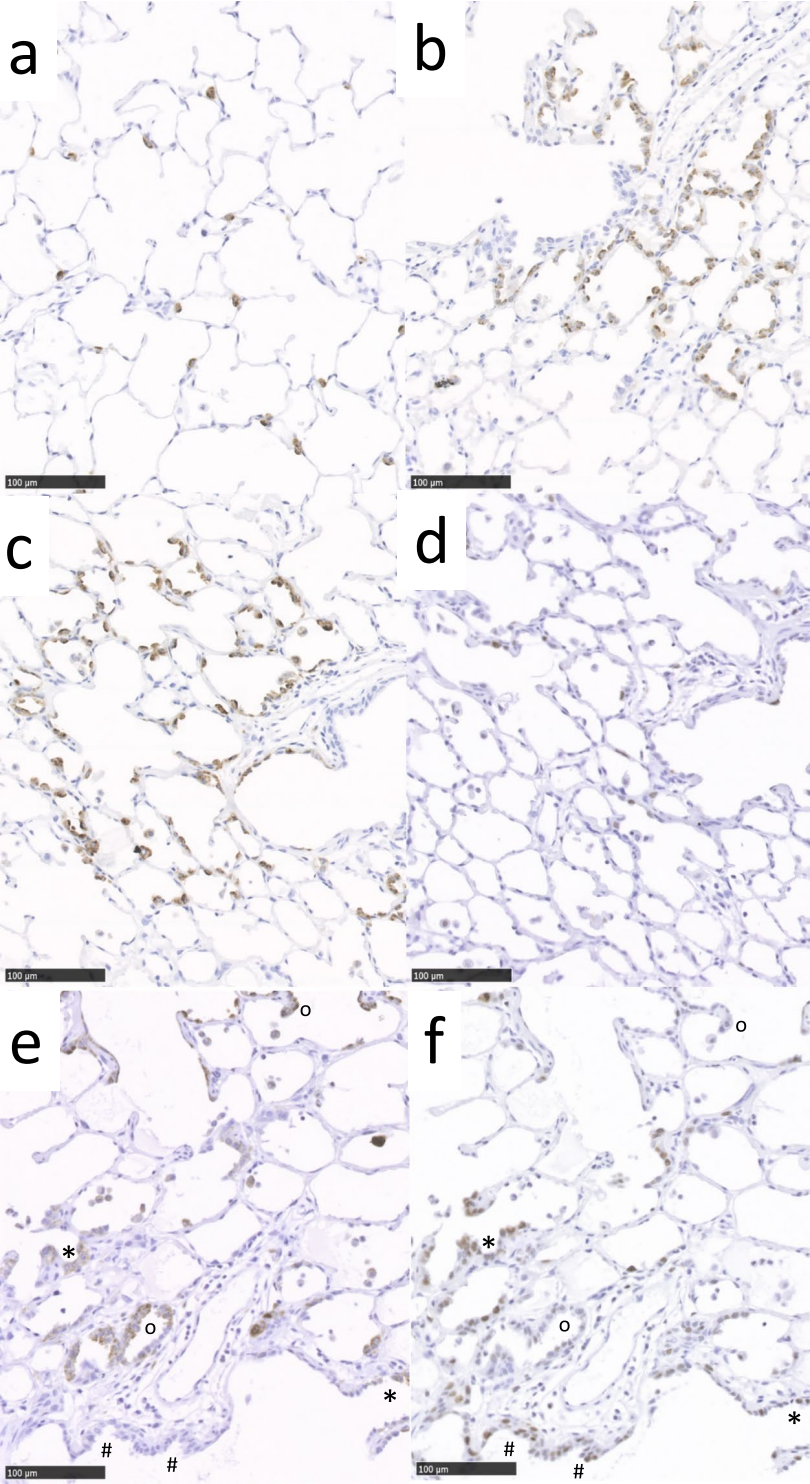
DC-LAMP and Ki67 expression associated with RILI in sheep. Panels (a) and (b) are photomicrographs of sections immunostained to depict DC-LAMP expression from radio-exposed (**b**) and non-radio-exposed (**a**) contralateral control lung (scale bar 100 μm). In control lung DC-LAMP was expressed by rounded cells in the alveolar corners assumed to be type II pneumocytes. These cells were evenly spaced throughout the distal lung parenchyma. In radio-exposed lung clusters of DC-LAMP-expressing cells could be clearly identified lining the walls of alveoli, with a concomitant reduction in expression in the alveolar region (not shown). Where clusters of DC-LAMP expressing cells could be identified (**c**), we assessed whether these cells were proliferating by immunostaining neighbouring serial sections (**d**) to depict the expression of Ki67 (scale bars 100 μm). In many instances the clusters of DC-LAMP expression were not associated with obvious cell proliferation. Indeed, the photomicrographs from serial sections in panels (e) depicting DC-LAMP expression (scale bar 100 μm), and (**f**) depicting Ki67 expression (scale bar 100 μm) illustrate the sometimes concordance between DC-LAMP and Ki67 expression (*), and the fact that DC-LAMP expression can occur in the absence of Ki67 expression (o), and vice versa (#).

DC-LAMP expression in non-radio-exposed lung was evident in large well-rounded cells most commonly positioned at the intersection of neighbouring alveolar walls (Fig. 6; panel (a)). Their appearance and position was consistent with their presumed identity as type II pneumocytes. These cells were regularly arrayed throughout the parenchyma. In contrast, in radio-exposed lung, DC-LAMP expressing cells were frequently found close together in linearly arranged clusters (Fig. 6; panels (b) and (c)). Whilst the areas between such clusters were largely devoid of the regular array of expression seen in the control lung, when cells were identified at the intersection of neighbouring alveolar walls, they appeared larger than those seen in the control lung sections. Clusters of positively labelled cells comprised contiguous, usually rounded but sometimes elongated or flattened, cells lining the alveolar walls.

**Figure 7:**
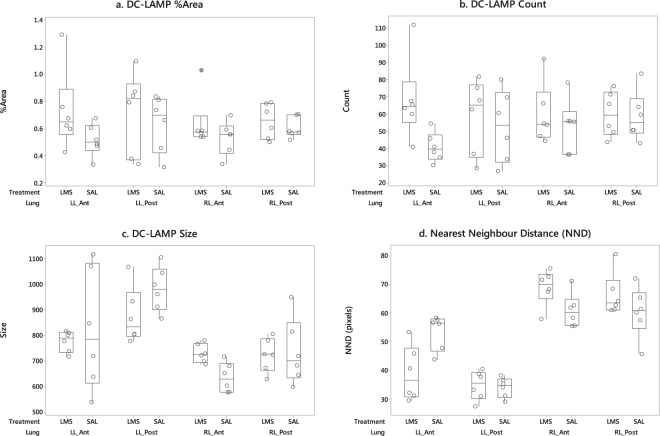
Quantification of DC-LAMP staining. Boxplot depicting data relating to (**a**) the percentage area of DC-LAMP expression, (**b**) the count of DC-LAMP expressing particles (DC-LAMP Count), (**c**) the average size of DC-LAMP-expressing particles (DC-LAMP size), and (**d**) the median Nearest Neighbour Distance (NND) as applied to DC-LAMP expressing cells present in parenchymal sections derived from the lungs of sheep previously exposed to radiation. The source of the sections is as described in the legend for Fig. 4, and in the material and methods.

The percentage area (%Area) of DC-LAMP positive staining, the number of DC-LAMP positive particles of a given size (150 pixels^2^-infinity), and the average particle size are depicted in Fig. 7a–c. Results from two-way analyses of variance conducted on the influence of the two independent variables (Lung, Treatment) on histochemical and immunohistochemical features is shown in Table 1. LAMELLASOME^TM^ pre-treatment significantly increased the number of DC-LAMP +ve cells throughout the lung (P = 0.047), and exposing lung to radiation caused a significant increase in the size of DC-LAMP +ve cells (P = 0.000). DC-LAMP +ve cells from castrated male sheep (793 μm^2^ [541–1117] median [range]) were significantly larger than those from female sheep (728 μm^2^ [579–961]). Further analysis of DC-LAMP expression involved calculating the nearest neighbour distances (NND) for positive-staining cells in each field. The boxplot in Fig. 7d depicts this metric. Two-way ANOVA (Table 1) indicated a significant lung effect (P = 0.000), a non-significant treatment effect (P = 0.554) and a significant interaction between these terms (P = 0.002) indicating that the lung effect depended on what treatment the sheep had received. Radiation exposure caused a decrease in DC-LAMP NND in directly exposed lung, and pre-treatment with LAMELLASOME^TM^ also was associated with a decrease in DC-LAMP NND in non-radio-exposed left lung (LL-Ant).

#### Ki67 expression

**Figure 8:**
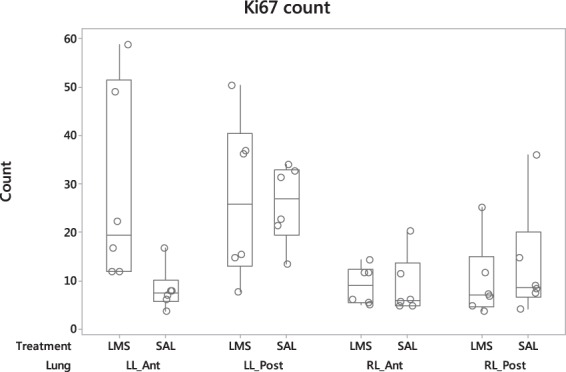
Quantification of Ki67 staining. Boxplot depicting data relating to the average number of Ki67-expressing cells (Ki67 count) present in image fields of lung parenchymal sections sourced from sheep previously exposed to radiation. The source of the sections is as described in the legend for Fig. 4, and in the material and methods.

Cells expressing Ki67 were only rarely observed in non-radio-exposed lung and were variously found in the septal walls, or alveolar airspaces. Proliferating cells were more commonly identified in radio-exposed lung. Occasionally these cells appeared to co-locate with cells expressing DC-LAMP (Fig. 6; panels (e) and (f)). Ki67-expressing cells could also be identified in perivascular fascia, and within the interstitium. The number of Ki67-positive cells is depicted in Fig. 8. Two-way ANOVA (Table 1) indicated a significant (P = 0.000) lung effect, a non-significant treatment effect (P = 0.221) and a significant interaction effect (P = 0.034) indicating that the lung effect depended on what treatment the sheep had received.

## Discussion

The consistent histopathological features associated with lung irradiation in this study, which developed within 37 days of the first exposure to radiation (within 23 days of the last exposure), were intra-alveolar oedema, alveolar fibrosis, interstitial pneumonia, and type II pneumocyte hyperplasia. Whilst observations of the earliest effects of radiation to human lungs are understandably unavailable, Gross (1977) in reviewing autopsy studies of humans dying of pneumonitis 4–12 weeks after radiotherapy also found alveolar septa thickened with oedema, cell infiltrates and the deposition of connective tissue, together with atypia, hyperplasia and desquamation of alveolar epithelial cells and the presence of hyaline membranes^34^. The early appearance of alveolar septal fibrosis is not an isolated finding. Indeed, Jennings & Arden (1962) found that alveolar septal fibrosis could be seen within a month of radiation exposure^35^, and Bennett *et al*. (1969) found alveolar septal fibrosis to be a prominent feature in five of seven patients with radiation pneumonitis who died between 40 and 95 days after completion of radiotherapy^36^.

While sheep share aspects of pathology with patients dying of radiation pneumonitis the sheep in the present study demonstrated no clinically overt adverse effect as a consequence of radiation exposure. We believe this reflects the coarse-fractionated dose regime and the relatively small volume targeted in the present study. Indeed it has previously been shown that sheep will develop radiation pneumonitis typical of that seen in humans given sufficient dose and lung volume targeted^37–41^. Unilateral single fraction high dose irradiation (30 Gy) of the sheep thorax produced radiation pneumonitis typical of the syndrome in humans at 4 weeks after irradiation^41^, and whole lung single fraction irradiation (15 Gy) of sheep resulted in the development of progressive dyspnoea three weeks after exposure; however animals were killed at 4 weeks precluding longer term assessment^38^. Some parallels and contrasts between these previous reports and our own experience are apparent. Guerry-force *et al*.^40^ also described interstitial and intra-alveolar oedema, interstitial accumulation of mononuclear cells, and epithelial injury to type I and II pneumocytes, but they found no evidence of interstitial fibrosis up to four weeks after exposure^40^. Considering our results and existing data, our conclusion is that sheep replicate many aspects of the early human response to lung irradiation, and that substantial pathology develops in sheep subjected to a radiotherapy regime (30 Gy/5F/2 wk), which bears resemblance to hypofractionated regimens routinely applied to patients with metastatic lung cancer, and some patients with locally advanced disease^42^. Our primary goal in this particular model was not to create clinical illness but rather to reproducibly irradiate a region of lung amenable to serial diagnostic sampling in which to assess the impact of an aerosolized synthetic drug.

Whilst peribronchial and peribronchiolar inflammatory cells, comprising mostly plasma cells and lymphocytes, were frequently identified in radio-exposed lung we found no evidence for radiation driving increased bronchial epithelial cytokine expression. Previous clinical studies which have assessed changes in plasma cytokine concentration during radiotherapy for lung cancer have demonstrated increased circulating TGF-β1^43^, IL-6 and IL-10^44^, and MCP-3, δMIP-1a, and IP-10^45^. Evidence for local lung production of inflammatory cytokines can be found in the studies of Barthelemy-Brichant *et al*.^46^ and Crohns *et al*.^47^. The former demonstrated increased IL-6 and TGF-β1 concentrations in BAL fluid^46^, and the latter a significant increase of BAL fluid IL-6 (but no change for TNFα, IL-1β or IL-12)^47^, associated with thoracic radiotherapy. Whilst correlations and contrasts can be found between these studies and our own it is nonetheless evident that radiation did not influence bronchial epithelial cytokine expression in this model at the time points assessed, a finding that is consistent with the observation that the bronchial epithelium is remarkably resistant to radiation doses that are routinely used in clinical brachytherapy^48,49^.

Alveolar fibrosis was a feature of the early pulmonary response to radiation in sheep pre-treated with saline. Alveolar mesenchymal fibroblasts are responsible for producing tropocollagen, the molecular component of collagen fibres, and the ground substance that fills the spaces between the cells and various fibres in the interstitial space. A particular population of differentiated fibroblasts comprise the myofibroblasts which are characterised by their expression of ASMA, as well as their ability to contract in a smooth muscle cell-like manner. Myofibroblasts play a fundamental role in alveologenesis^50^. In healthy lung sections ASMA expression is recognised at the tips of secondary septal crests, representing the cross-sectioned ridges running between the alveoli surrounding the alveolar ducts. The structure of the alveolar interstitial matrix is significantly compromised following radiation exposure. Central among the growth factors that co-ordinate matrix tissue re-modelling is TGF-β. This growth factor, which is ubiquitously expressed by all cells and tissues within the body, promotes extracellular matrix (ECM) deposition by stimulating different collagen, elastin, fibronectin and proteoglycan genes to produce ECM components. In addition to physical influences such as acidification or temperature changes TGF-β can be activated by proteases, by reactive oxygen species, or by interacting with thrombospondin or the αv-containing integrins (αvβ5, αvβ6, and αvβ8). Activated TGF-β can then interact with its receptors leading to phosphorylation of transcription factors Smad2 and/or Smad3 which in turn associate and form a complex with Smad4 before translocating to the nucleus to influence the transcription of target genes and the production of ECM components. In a rat model of radiation-induced lung injury, protein expression of integrin αvβ6, TGF-β1, TβRII, Smad3, and p-Smad2/3 was undetectable in the normal alveolar epithelium but increased in association with lung fibrosis six months after radiation exposure^51^. We opine that similar mechanisms are likely to be evoked in the context of the present model following sufficient time for fibrosis to be chronic and active, and this is an ongoing focus of our research.

We identified a significant positive association between ASMA expression and collagen deposition in lung sections from sheep exposed to radiation. A similar relationship is described in patients with pulmonary fibrosis^52,53^ and the implication arises that the ASMA-expressing myofibroblasts are also responsible for the increased production of collagen. This widely-held opinion has been recently challenged by evidence from a mouse model^54^ in which ASMA-directed deletion of αv integrins did not prevent lung fibrosis in a bleomycin mouse model, suggesting that other subpopulations of fibroblasts that don’t express ASMA (the majority) contribute to the integrin-mediated TGFβ activation and collagen production in this model. Other examples of increased ASMA expression in sheep lung include chronic lymphoid interstitial pneumonia in maedi-visna, a disease caused by the lentivirus maedi-visna virus (MVV) and characterised by lymphoid hyperplasia, hyperplasia of smooth muscle cells, alveolitis and fibrosis^29,55–58^, and mechanical ventilation-induced stretch injury in foetal sheep^59^. These diseases have markedly different aetiologies but can perhaps be linked with radiation injury through this common pathological response.

It is over 40 years since Kapanci *et al*. (1974) suggested that the presence of contractile interstitial cells in the alveolar wall might indicate that the alveoli themselves participate in the autoregulation of ventilation and perfusion^60^. Simple models of lung parenchyma at the acinar level have surface tension at the air-liquid interface on alveolar septa pulling the alveolar ducts radially outward, an effect counterbalanced by tissue forces (collagen, elastin and smooth muscle) in the alveolar entrance rings pulling the ducts radially inward^61^. The balance between these elements contributes to pressure–volume and geometric hysteresis during breathing^62^, and hence gas mixing and deposition in the pulmonary acinus. It is tempting to speculate that the increase in ASMA expression seen after radiation exposure is compensation for an increase in surface tension brought about by an impaired surfactant system.

Our analysis of DC-LAMP expression found that radiation exposure was associated with clustering and an increase in size of DC-LAMP-positive cells. Type II pneumocytes proliferate in response to injury and serve as progenitors for replacing lost or damaged type I pneumocytes lining the alveolar surface^63,64^. More recently it has been shown that adult, differentiated type I cells can themselves self-renew and give rise to type II cells^65^. Whilst some limited concordance between DC-LAMP and Ki67 staining did exist in the hyperplastic foci this was not a consistent finding and indeed it was perhaps surprising that evidence of proliferation was so scant in these areas. Type II cells are well-recognised to be early susceptible targets of radiation effects and we found that their reduced presence in their normal niches at alveolar corners was associated with an increase in size of the remaining cells found in these sites; altered timing of samples may provide additional information as to the role and period of post-radiation injury and proliferation. Whether the hypertrophy reflects perturbed or compensatory increase in function is unknown. Further, the mechanistic association between localised clusters of hyperplasia and the diffuse loss of type II pneumocytes is not intuitive. Whilst hyperplasia of type II pneumocytes is frequently observed in pathologic states^66^, these cells have also been described as the “defender of the alveolus”^67^, with their dysfunction lying at the heart of interstitial lung disease. Indeed, selective injury mediated through delivery of diphtheria toxin to transgenic mice expressing the diphtheria toxin receptor on these cells induced diffuse interstitial collagen deposition with patchy areas of more confluent scarring and associated alveolar contraction^68^. The underlying mechanisms remain to be elucidated but may reflect a homeostatic role for type II pneumocytes in the healthy lung. Recent evidence supports this notion as lung epithelial cells produce the anti-fibrotic mediator prostaglandin E2, which can mitigate the influence of TGF-beta^69^. Alternatively, type II pneumocytes can play an important role in mediating the pro-fibrotic influence of TGF-beta^70^. Li and colleagues (2011) demonstrated that loss of TGF-beta receptor signalling in these cells protected against bleomycin-induced fibrosis by inducing epithelial apoptosis, decreasing myofibroblast activation, and preventing a protease imbalance^70^. Senescence may also play a significant contribution to chronic lung injury following radiation. In a murine model of radiation-induced fibrosis, type II pneumocyte senescence occurred in a time and dose-dependent fashion following whole thorax irradiation, which was thought to reduce the stem cell compartment^71^. There is a complex set of pro-inflammatory, immunomodulatory and mitogenic cytokines that comprise the secretory profile of senescence (SASP), including TGF-β1, IL-1β, and IL-6 that have implications on RILI^72,73^. In a more recent murine study of radiation-induced lung fibrosis, rapamcyin effectively mitigated type II pneumocyte senescence, inflammatory cytokine expression, and collagen production^72^. Further study is warranted to determine whether the enlarged type II pneumocytes in the present study represent cells that have undergone radiation-induced early senescence. Our novel observation that DC-LAMP +ve cells from castrated male sheep were significantly larger than those from female sheep deserves further investigation to validate and explore the underpinning mechanisms.

Pre-treating sheep with nebulised LAMELLASOME^TM^ prior to each radiation exposure abrogated the increase in collagen seen in the PTV of sheep pre-treated with saline. LAMELLASOME^TM^ may have reduced the influence of reactive oxygen species and their impact on membrane permeability, thereby improving barrier function and preserving the physical and metabolic integrity of cells in the radiation path. Alternatively, or in addition, LAMELLASOME^TM^ may act more specifically to preserve type II cell function and the role of this cell in ‘managing’ the underlying interstitium. In support of a proposed type II cell effect LAMELLASOME^TM^ pre-treatment did significantly increase the number of DC-LAMP positive cells in the lung relative to sheep pre-treated with saline, which contributed to a non-significant trend (P = 0.067) towards an increase in the DC-LAMP area percentage. LAMELLASOME^TM^, in influencing the ability of type II cells to manage the interstitium, may subsequently decrease the proportion of myofibroblasts in this compartment in health, which would explain the significant treatment effect on ASMA. Until the differentially expressed genes and the respective networks associated with this effect are identified, the mechanism(s) involved must remain conjectural.

The literature holds many examples wherein preclinical small animal models of radiation-induced lung injury indicate that administration of putative radioprotectants have beneficial effects, both in the context of inflammation and/or fibrosis. However, as highlighted by Dabjan *et al*. (2016) the vast majority (95%) of reports investigating the radiation response of the mouse lung have involved whole lung or whole body radiation, with the remainder reporting the influence of radiation delivered to a fraction, usually half, of the lung^74^. Notably, in almost all of these reports the radioprotectant was delivered either systemically or orally. In the context of protecting the lung, aerosol delivery potentially allows the use of smaller doses that target more rapidly, and consequently avoid systemic side-effects. Despite these potential advantages there has, to our knowledge, been only one published report in which the mouse respiratory tract has been targeted by inhalation^75^. There has been no previous demonstration of radioprotection following aerosol delivery in a large animal model.

Whilst we observed no adverse effect relating to the delivery of LAMELLASOME™ in sheep it is appropriate to consider the likely safety profile of LAMELLASOME^TM^ in the context of its use as a clinical radioprotectant. LAMELLASOME™ in comprising saline, phosphatidylcholine, phosphatidylethanolamine, phosphatidylinositol and phosphatidyl serine, sphingomyelin and cholesterol has a very similar lipid profile to lung surfactant. LAMELLASOME™ has been tested clinically for ocular and oral indications, and has completed a safety and tolerability data package, including inhaled toxicology, with the latter studies indicating a no observed adverse effect level (NOAEL) dose of 65.5 mg/kg, equivalent to 4,585 mg for a 70 kg adult (A. Mclean, personal communication). These observations together with the knowledge that natural surfactants sourced from animals (poractant alfa and beractant) have been used widely in the clinic for decades suggest that the clinical risk of adverse effects associated with inhaled LAMELLASOME™ is low.

We sought an explanation for the observation that pre-treatment with LAMELLASOME^TM^ was associated with clustering of DC-LAMP positive cells and an increase in Ki67 count in non-radio-exposed left lung (giving rise to significant interaction effects). As conceptual mechanisms for such unilateral lung effects are not obvious we hypothesised that the proximity of the anterior blocks to the cranial margin of the PTV might be a factor in dictating this difference between the treatment groups. Our strategy for selecting these anterior blocks was as follows: The block containing the cranial margin of the PTV was identified. Progressing cranially, its immediate neighbour was disregarded and the next block along selected as LL (or RL)_Ant. As this procedure was consistent it was assumed that any variation in the spatial relationship between the selected blocks and the cranial margin of the PTV would be randomly spread between the saline and LAMELLASOME^TM^ groups. However, when we retrospectively examined these distances we discovered that there was a significant difference between the groups in terms of the distance of the “Ant” section from the cranial margin of the PTV – the LAMELLASOME^TM^ sections were approximately 7 mm closer (data not shown). Further, when the relationship between LL_Ant and RL_Ant DC-LAMP NND and Ki67 cell counts was expressed as absolute difference (LL-RL), and fold-change (1 + ((LL-RL)/RL) respectively, and compared to the distance of the section from the cranial edge of the PTV, significant correlations were found which would appear to explain the observation that pre-treatment with LAMELLASOME^TM^ was associated with a decrease in DC-LAMP NND and an increase in Ki67 count in non-radio-exposed left lung (data not shown). This suggests a ramped decline in the biological effect of radiation (at least in terms of DC-LAMP cell clustering and cell proliferation) that extends beyond the margins of the PTV in this model. As lung is a heterogeneous structure and photons are most predictable when traversing through solid tissue, these changes likely reflect the radiation “scatter” to adjacent lung or bystander effects, thus creating a dose-gradient immediately cranial to the PTV.

Currently amifostine is the only drug approved by the U.S. Food and Drug Administration for protection from radiation. Administered as an inactive prodrug it is dephosphorylated by alkaline phosphatase in the normal endothelium to form an active thiol which scavenges free radicals, induces cellular anoxia and protects DNA. Although several non-randomized clinical trials have demonstrated that amifostine can reduce the severity of lung injury after radiotherapy^76,77^ its use has been generally limited to head and neck cancer patients because of its potential severe side effect profiles. Finding new ways of mitigating RILI with minimal adverse effects is recognised as an urgent need. Our study, in demonstrating the mitigating influence of LAMELLASOME^TM^ on RILI, is the first to use a large animal platform to investigate aerosolised lung protection following fractionated radiotherapy.

The lack of adverse effect, the ability to target selected lung volumes using fractionated regimes, the ability to follow longitudinal changes in the same animal, and the utility of comparing within-animal responses between radio-exposed and non-exposed lung, all contribute to a robust and highly tractable platform with which to explore aspects of the RILI response and investigate the impact of radioprotectant approaches. We believe that our chosen delivery strategy also had an important bearing on our findings. In addition to the aforementioned dose, targeting and side-effect issues, administering radioprotection via aerosol offers many of the same advantages that are cited in the context of mass vaccination by the same route – namely ease and speed of application by nonmedical personnel, non-invasiveness which results in greater social acceptance, reduced risk of cross-contamination of blood-born infectious agents, diminished medical waste, and potentially lower costs^78,79^. Further studies will examine the ability of aerosolized surfactant to alter pathological, molecular and gene expression changes following altered dosing and fractionation schemes with prolonged assessment periods.

## Electronic supplementary material

Supplementary methods

## Data Availability

The datasets generated and analyzed during the current study are available from the corresponding author on reasonable request.
