# Supplementary material for: Nebulisation of synthetic lamellar lipids mitigates radiation-induced lung injury in a large animal model
Source: Sci Rep. 2018 Sep 6;8:13316. doi: 10.1038/s41598-018-31559-3 (PMC6127301; doi:10.1038/s41598-018-31559-3)
Supplement: Supplementary file 1 — Supplementary methods [file 41598_2018_31559_MOESM1_ESM.pdf]

Supplementary methods

Nebulisation of synthetic lamellar lipids mitigates radiation-induced lung injury in a large animal model

David Collie[1], John T Murchison[2], Steven H Wright[1], Alec McLean[3], Lynsey Howard[3], Jorge del-Pozo[1], Sionagh Smith[1], Gerry McLachlan[1], Jessica Lawrence[1], Elaine Kay[1], Tobias Schwarz[1], Magdalena Parys[1]

[1] The Roslin Institute and Royal (Dick) School of Veterinary Studies, University of Edinburgh, Edinburgh, United Kingdom

[2] Edinburgh Imaging, College of Medicine and Veterinary Medicine, University of Edinburgh, Edinburgh, Scotland, United Kingdom

[3] Lamellar Biomedical Ltd, Eurocentral, Holytown, Scotland, United Kingdom

Details of immunohistochemistry protocols

ASMA - Non-specific binding was blocked with 10% Normal Goat Serum (Sigma G9023) in PBS + 0.5% Tween 80. Primary antibodies, monoclonal anti α-smooth muscle actin (Sigma A2547) and normal mouse IgG isotype control (Sigma M5284) were diluted to 1µg/ml in blocking buffer and incubated for 30 minutes at room temperature. Detection was achieved using biotinylated goat anti mouse IgG (Vector BA-2001) and Streptavidin peroxidase polymer (Sigma S-2438) followed by DAB substrate (Vector SK-4100) with Haematoxylin counterstain.

Ki67- Non-specific binding was blocked with 3% BSA (Sigma A3733) in PBS + 0.05% Tween 20. Primary antibodies Monoclonal anti Ki67 clone MIB-1 (Dako M7240) and Normal Mouse IgG isotype control (Sigma M5284) were diluted to 1µg/ml and incubated for 45 minutes at room temperature. Detection was achieved using biotinylated goat anti mouse IgG (Vector BA2001) and Streptavidin peroxidase polymer (Sigma S-2438) followed by DAB substrate (Vector SK-4100) and Haematoxylin counterstain.

DC-LAMP- Non-specific binding was blocked with 4% normal rabbit serum (Sigma R9133) in PBS + 0.2% Tween 80. Primary antibodies DC-LAMP/CD208 (2BScientific DDX0191P-50) and Normal Rat IgG isotype control (Serotec MCA1125R) were diluted to 2.5µg/ml in blocking buffer and incubated overnight at 4°C. Detection using biotinylated goat anti rat IgG (Vector BA4001) and Streptavidin peroxidase polymer (Sigma S-2438) followed by DAB substrate (Vector SK-4100) and Haematoxylin counterstain.

Gene	Pre incubation	Amplification	Melting curve
Ovine ATPase and IL1 beta	95°C for 15 minutes	50 cycles of<br>94°C 15 seconds<br>52°C 30 seconds<br>72°C 30 seconds<br>80°C 5 seconds	95°C 5 seconds ramp 4.4<br>40°C 1 minute ramp 2.2<br>97°C continuous 0.11<br>72°C 10 minutes ramp 2.2
Ovine TGF beta	50°C 2 minutes<br>95°C 8.5 minutes	40 cycles of<br>95°C 15 seconds<br>60°C 1 minute	95°C 1 minute ramp 4.4<br>55°C 1 minute ramp 2.2<br>97°C continuous 0.11
Ovine IL8	45°C 30 minutes<br>95°C 10 minutes	40 cycles of<br>94°C 20 seconds<br>55°C 30 seconds<br>72°C 30 seconds	95°C 5 seconds ramp 4.4<br>55°C 1 minute 2.2<br>95°C continuous 0.29
Ovine ACTB	95°C 10 seconds	40 cycles of<br>95°C 15 seconds<br>60°C 1 minute	95°C 5 seconds ramp 4.4<br>60°C 1 minute ramp 2.2<br>97°C continuous 0.11

Table S1 qPCR conditions

PCR conditions for the amplification of transcripts from ovine ATPase, IL1 beta, TGF-beta, IL8 and ACTB.

Gene	Forward Primer 5’-3’	Reverse Primer 5’-3’	Reference
Ovine ATPase	GCTGACTTGGTCATCTGC	CAGGTAGGTTTGAGGGGATAC	24
Ovine ACTB	CCAAGGCCAACCGTGAGA	AGCCTGGATGGCCACGT	25
Ovine TGF beta	CTGAGCCAGAGGCGGACTAC	TGCCGTATTCCACCATTAGCA	26
Ovine IL1 beta	CCCATTAATGAAGTGATGGC	CTAGGGAGAGAGGGTTTCCA	24
Ovine IL8	CACTGCGAAAATTCAGAAATCATTGTTA	CTTCAAAAATGCCTGCACAACCTTC	27

Table S2 qPCR primer sets

The primers used in this study
